# Profile Comparer Extended: phylogeny of lytic polysaccharide monooxygenase families using profile hidden Markov model alignments

**DOI:** 10.12688/f1000research.21104.1

**Published:** 2019-10-31

**Authors:** Gerben P. Voshol, Peter J. Punt, Erik Vijgenboom

**Affiliations:** 1 Department of Microbial Biotechnology and Health, Insitute of Biology Leiden, Leiden, 2333BE, The Netherlands; 2 Dutch DNA Biotech B.V., Utrecht, 3584CH, The Netherlands

**Keywords:** LPMO, HMM, Hidden Markov Model, Lytic Polysaccharide Mono-oxygenase, phylogeny

## Abstract

Insight into the inter- and intra-family relationship of protein families is important, since it can aid understanding of substrate specificity evolution and assign putative functions to proteins with unknown function. To study both these inter- and intra-family relationships, the ability to build phylogenetic trees using the most sensitive sequence similarity search methods (e.g. profile hidden Markov model (pHMM)–pHMM alignments) is required. However, existing solutions require a very long calculation time to obtain the phylogenetic tree. Therefore, a faster protocol is required to make this approach efficient for research. To contribute to this goal, we extended the original Profile Comparer program (PRC) for the construction of large pHMM phylogenetic trees at speeds several orders of magnitude faster compared to pHMM-tree. As an example, PRC Extended (PRCx) was used to study the phylogeny of over 10,000 sequences of lytic polysaccharide monooxygenase (LPMO) from over seven families. Using the newly developed program we were able to reveal previously unknown homologs of LPMOs, namely the PFAM Egh16-like family. Moreover, we show that the substrate specificities have evolved independently several times within the LPMO superfamily. Furthermore, the LPMO phylogenetic tree, does not seem to follow taxonomy-based classification.

## Introduction

Renewable feedstocks, such as wheat straw, rice straw and other agricultural waste residues are used by the bioindustry for the production of sugars and value-added products. One of the first steps in this process is the enzymatic breakdown of these raw materials into smaller building blocks. For this, hydrolytic enzyme cocktails are extensively used. However, some biopolymers are resistant to complete enzymatic degradation by available enzyme cocktails. Lytic polysaccharide monooxygenases (LPMOs) are a relatively new class of metalloenzymes that can perform oxidative cleavage and aid breakdown by conventional hydrolytic enzymes ( Harris *et al.*, 2010; Vaaje-Kolstad *et al.*, 2010).

Currently there are seven families of LPMOs defined in the Carbohydrate–Active Enzymes database (CAZy) ( Lombard *et al.*, 2014), namely the auxiliary activity families AA9 (formerly GH61), AA10 (formerly CBM33), AA11 ( Hemsworth *et al.*, 2014), AA13 ( Vu *et al.*, 2014), AA14 ( Couturier *et al.*, 2018), AA15 ( Sabbadin *et al.*, 2018; Voshol *et al.*, 2017) and AA16 ( Filiatrault-Chastel *et al.*, 2019; Voshol *et al.*, 2017). Although identifying members belonging to these known families is relatively easy, it is more difficult to identify members belonging to potentially novel LPMO families ( Lo Leggio *et al.*, 2015), given the very low level of overall sequence similarity between LPMO families. Therefore, we developed a profile hidden Markov model (pHMM) and used it to mine several genomes for new LPMO families ( Voshol *et al.*, 2017). pHMM-sequence searches are sensitive enough to identify putative LPMOs, but they are not suitable to establish the evolutionary relationship between these LPMOs. For example, a pHMM build from an alignment of AA13s was only able to identify AA13s ( Lo Leggio *et al.*, 2015) indicating that a more sensitive approach is necessary to build a phylogeny for all LPMOs.

pHMM-pHMM alignments are the most sensitive for this purpose ( Sadreyev & Grishin, 2008; Söding, 2005). In 2017, Huo and colleagues developed a pHMM phylogentic tree approach and used it to study the evolutionary relationship of CAZy protein families with pHMM-pHMM alignments (pHMM-tree; Huo *et al.*, 2017). Unfortunately, due to the exponential time required for generating the distance matrix and the tree, the number of pHMMs which can be included in the phylogenetic tree is limited (max 500). Therefore, this program is not applicable to study the relationship of proteins within large families.

In this study we apply both pHMM-sequence searches and pHMM-pHMM alignments to gain a deeper understanding of LPMO domain organization and phylogeny. To overcome the limitations of pHMM-tree, we extended the original Profile Comparer program (PRC; Madera, 2008) for the construction of large pHMM phylogenetic trees (>1800 HMMs) and added several additional capabilities. The resulting program, named PRCx (PRC eXtended) is several orders of magnitude faster than pHMM-tree and was used to reveal both the inter- and intra-family LPMO evolutionary relationship. Moreover, using PRCx, we were also able to reveal a previously unknown distantly related member of the LPMO superfamily.

## Methods

**Figure 1.**
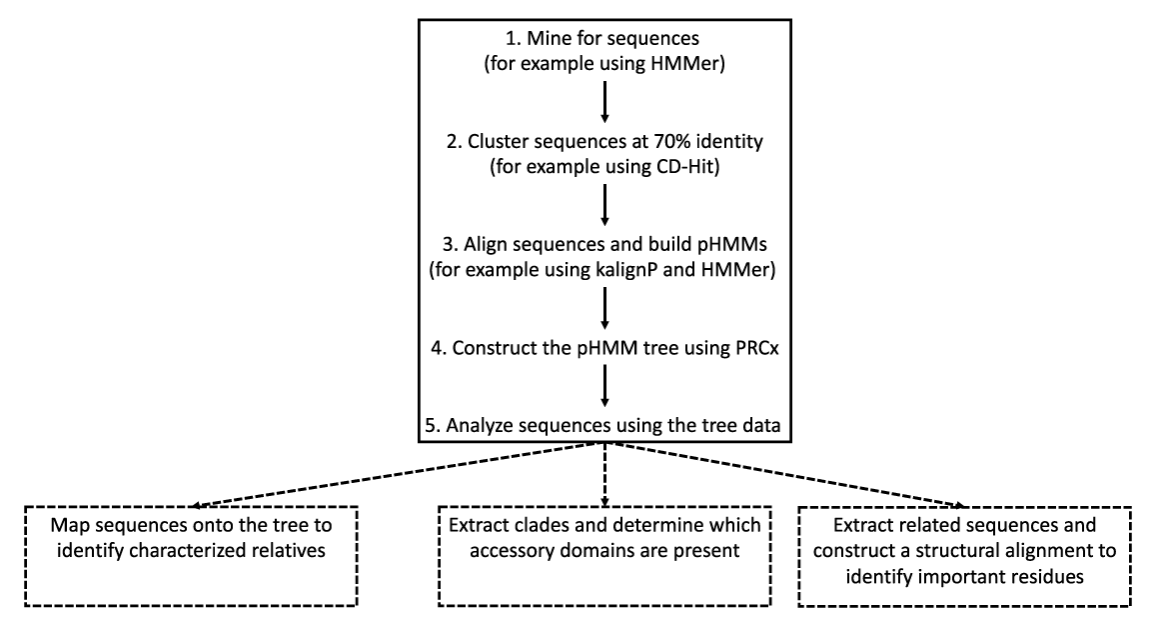
Flow chart indicating the steps in using PRCx. The steps are as follows. Create a sequence database, cluster sequences and construct alignments from them. Convert these alignments to pHMMs and construct the tree. The resulting tree can be used for example to identify relatives, extract sequences from a clade and mine determine their accessory domains or perform structural alignments to identify important residues.

**Figure 2.**
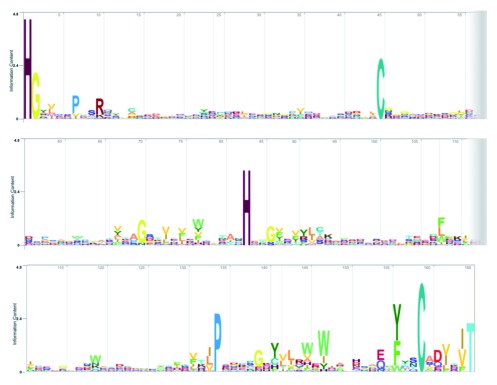
Logo of the core LPMO pHMM after 10 search iterations of the UniProt database (created using the HMMER web server). The height of the letter indicates the information content (level of conservation and the number indicates the position in the pHMM.

To create the initial LPMO dataset (See Figure 1), the UniprotKB database (downloaded on 18-10-2017) was searched for 10 iterations using a truncated version (containing only the “core” LPMO domain, see Figure 2) of the previously published pHMM ( Voshol *et al.*, 2017). This core LPMO pHMM has a total model length of 165, starting at the N-terminal histidine, that makes up part of the histidine brace, up to a relatively well conserved threonine. With the aim to analyze proteins related to LPMOs an E-value of 1 was used. It is possible to extend the dataset with another ~20% using an E-value of 1000 at the expense of increasing the number of unrelated hits ( Wistrand & Sonnhammer, 2005).

After generating the initial dataset, the taxonomic distribution and the presence of accessory domains were analyzed using the HMMER web server ( Potter *et al.*, 2018). The sequences were retrieved and a non-redundant dataset was created by clustering sequences at a 100% sequence identity using the CD-HIT toolset ( Fu *et al.*, 2012; Li & Godzik, 2006). The non-redundant dataset was subsequently clustered at 70% sequence identity and sequences contained within those clusters were grouped into their respective fasta files. Fasta files containing two or more sequences where aligned using the kalignP alignment program ( Lassmann *et al.*, 2009; Shu & Elofsson, 2011) and pHMMs were built using HMMer 3.0 ( Eddy, 2011). This resulted in 1828 pHMMs and 2296 singletons (sequences which did not cluster at 70% identity with any other sequence). PHMMs from dbCAN2 and PFAM protein families were downloaded from their respective web servers ( El-Gebali *et al.*, 2019; Yin *et al.*, 2012; Zhang *et al.*, 2018). PRCx was used to search for distantly related LPMO PFAM protein families that were used as an outgroup during the tree building stage (see *Results* for more details).

### Implementation

Several new features were added to the original PRC program ( Madera, 2008), including the ability to (i) use HMMer3.0 pHMM files, (ii) build pHMM using single or aligned fasta files, (iii) speed up pHMM-pHMM searches using prefiltering and (iv) generate a PHYLIP compatible distance matrix and associated UPGMA Newick formatted phylogenetic tree ( Felsenstein, 1989). 

The original PRC program has the ability to, amongst others, load SAM3, HMMer2 and PSI-Blast profile files ( Madera, 2008). However, since the release of the original PRC program in 2008, a new version of HMMer was released in 2011 ( Eddy, 2011). Soon thereafter, public databases such as PFAM and dbCAN updated to the newer HMMer version. Since this format is used so extensively, we added support for HMMer3.0 pHMM files to PRC.

To facilitate both pHMM building and fast prefiltering, support for sequence context-specific pseudocounts was added. The idea behind context-specific pseudocounts is that the local environment around an amino acid determines what mutations can occur at that particular amino acid location ( Overington *et al.*, 1992). This rationale has been applied in numerous programs to increase the sensitivity of protein-protein alignments ( Gambin *et al.*, 2002; Huang & Bystroff, 2006; Jung & Lee, 2000). For PRCx we implemented the context-specific pseudocount method for the context-specific BLAST program ( Biegert & Söding, 2009).

An additional advantage of implementing support for context-specific libraries is the ability to reduce the amino acid probability vectors of a pHMM to a discretized alphabet. This was achieved by the same method as used by HHblits to translate the amino acid profiles to 219 distinct letters ( Remmert *et al.*, 2011). Subsequently a mutational substitution matrix was calculated and used together with a fast implementation of the Single-Instruction-Multiple-Data Smith-Waterman algorithm ( Zhao *et al.*, 2013; Remmert *et al.*, 2011).

The final noteworthy feature is the ability to create a distance matrix by comparing all the pHMMs in a library of pHMMs against each other and determining the simple co-emission score ( Madera, 2008). This score is converted to a distance score identical to the algorithm as used by the pHMM-tree program ( Huo *et al.*, 2017). The resulting distance matrix is saved in a PHYLIP-compatible file and used to build an unweighted pair group method with arithmetic mean (UPGMA)-based phylogenetic tree. This means that given identical input pHMMs, trees generated using pHMM-tree and PRCx are identical. This was manually validated for a tree generated using the top 248 pHMMs out of the total 1828 pHMMs generated using both PRCx and pHMM-tree. In our implementation, the most time-consuming step was the UPGMA clustering. Therefore, we adapted the fast O(n ^2^) algorithm as implemented in the MUSCLE and Clustal Omega alignment programs ( Edgar, 2004; Sievers *et al.*, 2011).

### Operation

The PRCx program was developed and tested using both GNU/Linux (Ubuntu version 18.04) and MacOSX (version 10.14.5). The computer system used for testing contained an Intel Core i5 with 8 GB of memory.

## Results

The initial sequence dataset was created by iteratively searching the UniprotKB database using the Jackhmmer program and our previously published LPMO pHMM ( Johnson *et al.*, 2010; Voshol *et al.*, 2017). After 10 iterations, 12819 non-redundant putative LPMO sequences were identified. The resulting refined pHMM ( Figure 2) clearly shows several residues that have a high informational content (i.e. conserved residues). Not surprisingly, these residues include the two histidines that form the essential copper binding histidine brace ( Aachmann *et al.*, 2012; Chaplin *et al.*, 2016; Gudmundsson *et al.*, 2014; Hemsworth *et al.*, 2013). Another conserved feature is the N/Q/E-x-F/Y/(W) motif, which was previously used to mine for novel starch active LPMOs ( Vu *et al.*, 2014). Finally, there are two conserved cysteines and a proline. The proline is located distal from the active site therefore it is most likely important for structural reasons ( Voshol *et al.*, 2017).

### Taxonomic occurrence and domain organization

**Figure 3.**
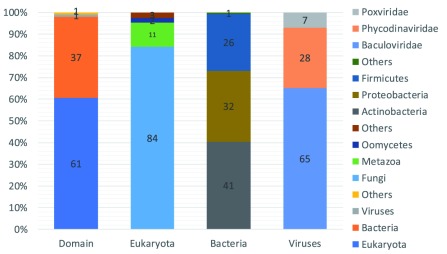
Taxonomic occurrence of LPMO sequences mined. From left to right, the first bar shows the distribution of the sequences according to their Domain, Eukaryota, Bacteria, Viruses, Others, indicated in percentages of total sequences. The following three bars indicate the distribution (in percentages) of sequences as a function of the total number of sequences in the Domain (indicated below the bar).

**Figure 4.**
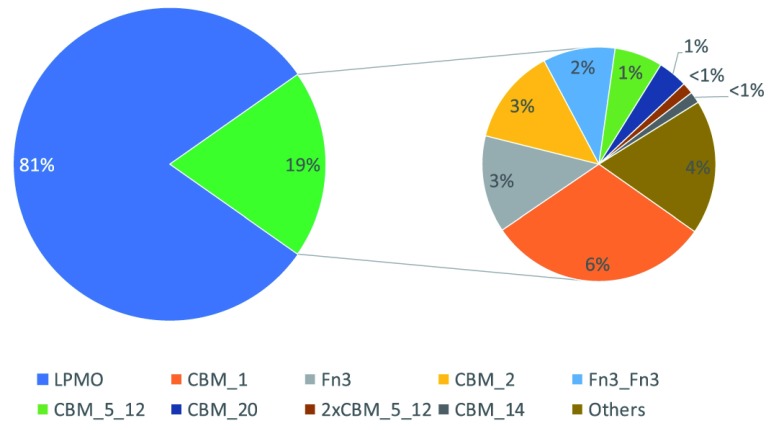
LPMO sequences with known accessory domain. Indicated is the percentage of LPMO sequences that have a known accessory domain (left, green). Those with a known accessory domain are indicated in more details (right) with their occurrence in percentage of total LPMO sequences and rounded to the nearest full percentage.

After the initial dataset was created, the taxonomic occurrence and domain organization were analyzed using the HMMER web server ( Potter *et al.*, 2018). The dataset mainly contains sequences belonging to the domains of Eukaryota and Bacteria (98%) ( Figure 3). Within the domain of Eukaryota, Fungi are by far the largest contributor of LPMO sequences (84%). This is in line with the hypothesis that Fungi play a major role in the global carbon cycle and contain a large repertoire of carbohydrate-degrading enzymes ( Benocci *et al.*, 2017). Actinobacteria, proteobacteria and Firmicutes contribute most of the LPMO sequences (99%) within the domain of Bacteria. The sequences identified in viruses are predominantly from the Baculoviridae (65%) and Phycodinaviridae (28%). The only two Archaeal LPMO sequences that were found, both belong to the Euryarchaeota. Out of all the LPMO sequences identified, only 19% have known accessory, mainly carbohydrate binding, domains ( Figure 4).

### Phylogenetic tree

To gain a better understanding of LPMO evolution, Book *et al.* (2014) created two phylogenetic trees, one for the AA10s and one for the AA9s. With their approach, they were able to show that there are different clades within these two families and each clade has evolved a specific substrate and oxidation preference (e.g. C1, C4, C1/C4). However, their approach is not sensitive enough to show the relation between the different families of LPMOs, therefore we undertook the construction of a comprehensive phylogenetic tree using the sensitivity of pHMM alignments.

Before building the LPMO tree, we searched PFAM for related families of the core LPMO HMM to find an appropriate outgroup (starting point of the tree). As expected, the PFAM LPMO_10 (PF03067) and GH61 (PF03443) families were identified as close relatives. Surprisingly, we were also able to identify one distantly related family, namely the PFAM Egh16-like family, formerly known as DUF3129 (PF11327; available from http://pfam.xfam.org). The homology between the Egh16-like family and the LPMO family is in part due to the histidine located at the third position of the PFAM HMM, which in the LPMO family is part of the histidine brace. It should be noted that the Egh16-like family HMM is presumably based on an incorrectly predicted signal peptide cleavage site, resulting in the conserved histidine not being the first residue of the PFAM model. When examining several sequences within the Egh16-like family, the latest version of SignalP predicts the signal peptide cleavage site right before the histidine ( Almagro Armenteros *et al.*, 2019). Unlike the LPMO family however, the Egh16-like family does not appear to have a second histidine (forming the histidine brace), but instead contains a conserved aspartic acid. The Egh16-like family is restricted to Fungi and proteins within this family might play an important role in pathogenic fungi in the early stages of plant and insect infection ( Xue *et al.*, 2002).

**Figure 5.**
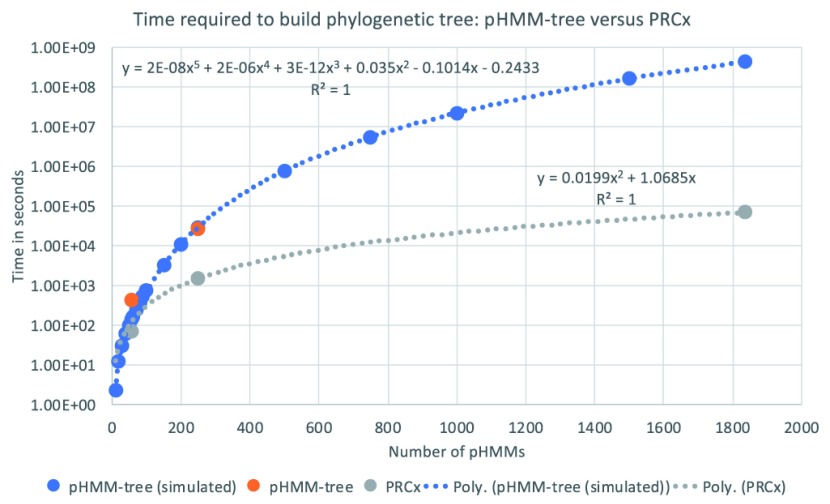
The runtime of pHMM-tree ( Huo *et al.*, 2017), both simulated (based on pHMM-tree article in blue) and real (in orange), versus that of PRCx (gray). The X-axis indicates the number of pHMMs in the tree and the Y-axis is the runtime in seconds. For example, building a tree containing 248 pHMMs with pHMM-tree took 27,059 seconds (~7.5 hours), while building the same tree with PRCx took 1504 seconds (~25 minutes). The blue and grey lines are the estimated trend lines that best fits the data for pHMM-tree and PRCx, respectively.

After the outgroup was identified, the LPMO phylogenetic tree was built as follows. The original nonredundant dataset of 12,819 sequences was clustered at 70% homology (leaving 2296 sequences as singletons) and sequences contained within where aligned and used to build HMMs. Initially a small tree was constructed, containing a subset of 248 HMMs, using the pHMM-tree program ( Huo *et al.*, 2017). This process took 7.5 hours. Extrapolating this amount of time to the time required to make the entire tree (>1800 HMMs), would result in a tree construction time of 14 years. This is in line with the original paper describing pHMM-tree and its algorithm ( Huo *et al.*, 2017). As an alternative, it was decided to extend PRC to be able to make simple UPGMA phylogenetic trees. This resulted in PRCx, which was able to build the small tree (248 HMMs) in 0.5 hours and the final tree in approximately 20 hours. Which is a 15-6000x speed improvement versus the original pHMM-tree method ( Figure 5).

**Figure 6.**
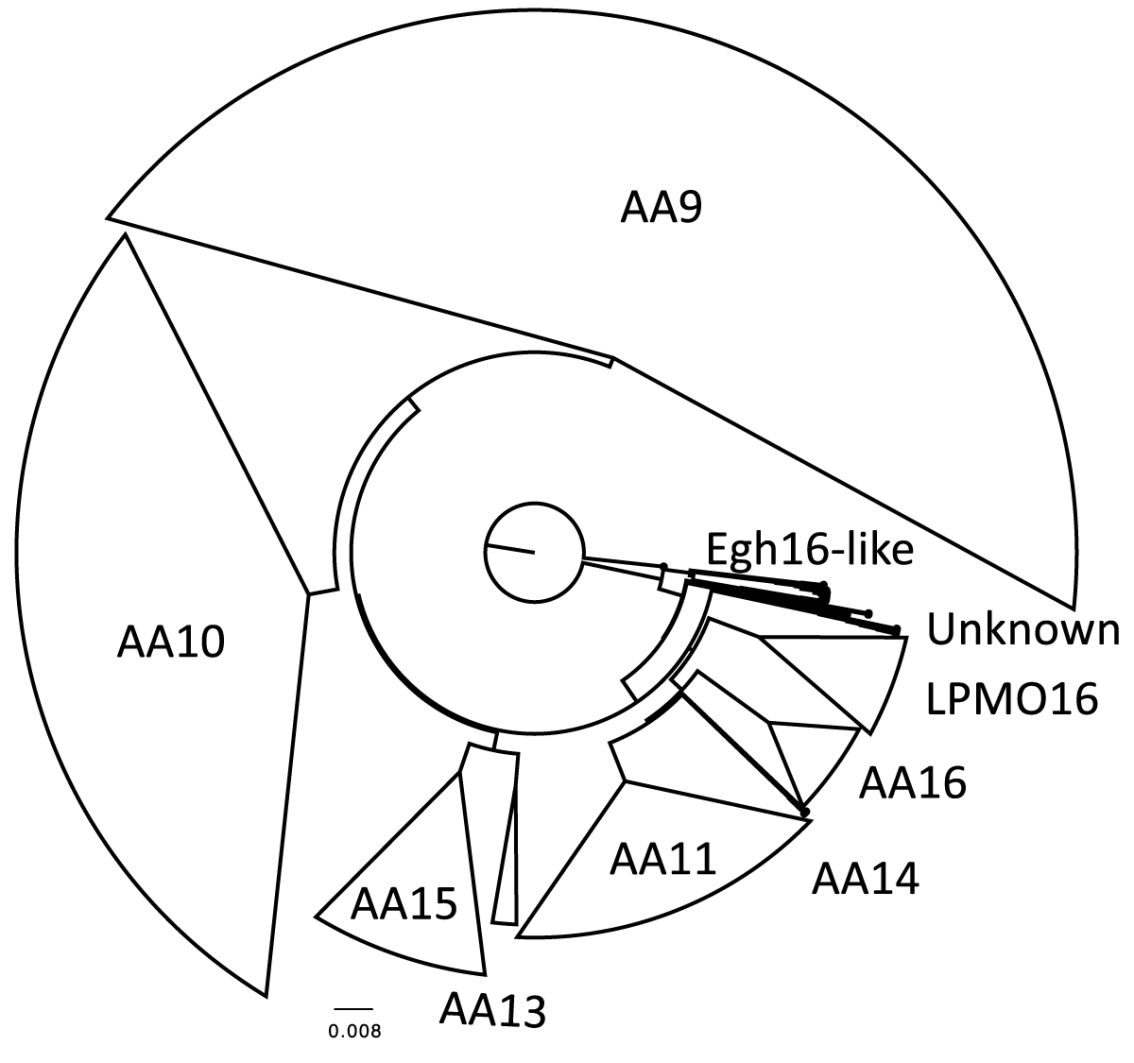
Simplified LPMO phylogenetic tree and relative abundance of LPMO families. The initial non-redundant dataset was clustered at 70% sequence homology and each cluster resulted in a single alignment. PHMMs were build and a UPGMA tree was constructed using PRCx. The phylogenetic tree was subsequently rooted using the Egh16-like family as an outgroup.

The resulting tree was rooted using the Egh16-like family as an outgroup. A simplified representation is shown in Figure 6 and the entire tree is available as a searchable PDF (Figure S1) with sequence data (Table S1) (see *Extended data*; Voshol *et al*., 2019a). As can be seen from the tree, the AA9s are by far the largest family (41%), followed by AA10s (27%), AA11s (14%), AA15s (7%), AA16s (4%), LPMO16s (4%), AA13s (1%) and AA14s (<0.5%). An additional 2% of HMMs branch off early in the LPMO tree before any of the known or putative LPMO families. The earliest branch splits into two branches, namely one strictly containing Egh16-like members and another which splits further and contains PFAM DOMON/EGF and LPMO_10 domain-containing sequences. The DOMON domain might play a role in metal or sugar binding and is often associated with redox enzymes ( Iyer *et al*., 2007). A more detailed biochemical understanding of what the Egh16-like family does will shed a better light upon the possible relation of the Egh16-like, LPMO_10, DOMON and EGF domains.

When moving up the tree the first large branch contains the LPMO16s which were previously identified as putative LPMOs while mining genomes of filamentous Fungi (represented by An07g08250 in *Aspergillus niger*) ( Voshol *et al.*, 2017). This family is related to the AA16s ( Filiatrault-Chastel *et al.*, 2019; Voshol *et al.*, 2017), AA14s ( Couturier *et al.*, 2018) and AA11s (( Hemsworth *et al.*, 2014). This suggests that the common ancestor of this branch evolved not only to oxidize cellulose (AA16s), but also xylan (AA14s) and even chitin (AA11s). A similar observation can be made for the next branch, which contains the AA15s and the AA13s. The AA15s were first identified in 2017 and later it was shown that they have the ability to cleave cellulose or chitin ( Sabbadin *et al.*, 2018; Voshol *et al.*, 2017). The AA13s were identified and characterized in 2014 and can cleave starch ( Vu *et al.*, 2014). Taken together, this suggests that ancestral LPMOs have evolved multiple times to oxidize a diverse range of substrates. The tree is completed with the large AA10 and AA9 family of LPMOs. The AA10 contains LPMOs which can cleave both cellulose and chitin, while the AA9 family contains members which can cleave cellulose or xylan. Similar to the observations by Book *et al.* (2014), clades within the AA9 and AA10 family appear to have a specific substrate and oxidation preference. However, only a tiny percentage of LPMOs have been characterized and even in these cases the measured enzyme activity may have been misinterpreted ( Eijsink *et al.,* 2019). This makes drawing general conclusions on functionality somewhat preliminary.

On closer examination, the AA9 clade also contains LPMOs which have either an arginine or a lysine instead of the N-terminal histidine ( Yakovlev *et al.*, 2012). An arginine containing LPMO has recently been characterized, but no activity was identified ( Frandsen *et al.*, 2019). The place of these LPMOs present in node 726 and 650 suggest that these LPMOs evolved relatively recent from “normal” histidine-containing AA9 LPMOs. It would therefore be interesting to see whether restoring the arginine or lysine to a histidine will result in active LPMOs.

Taxonomically, the LPMO subfamilies as we have classified them with PRCx, have a peculiar distribution different from either their substrate or taxonomic based classification (see Table S1). The subfamilies, AA9, AA11, AA13 and AA14 are mostly found in Fungi (>90% of LPMO sequences), the AA16 are found in both Fungi (82%) and Oomycetes (12%), while the AA10 are almost exclusively bacterial (99%) and the AA15 are mainly found predominantly in Metazoa (95%). The recently discovered LPMO16 are mostly found in Fungi (78%), but are also found in Metazoa (4%) and Oomycetes (6%). This observation suggests that LPMOs have found their true functional diversity in the fungal kingdom.

## Use cases

After constructing the phylogenetic tree, it is possible to use it in several ways. For example, it is possible to search an unknown sequence against the pHMMs used for the tree building and discover to which LPMO subfamily and specific branch this protein belongs. This might give an indication of substrate specificity and oxidation preference that the newly discovered protein has.

It is also possible to extract sequences or pHMMs from the tree that belong to a specific LPMO subfamily or clade. These can subsequently be analyzed for the presence of specific accessory domains or domains of unknown function. This might also give an indication of localization or substrate preference. For example, after extracting all the AA15 pHMMs and searching them against the PFAM database using PRCx, it appears that some of the members have a fasciclin domain. This domain may be involved in cell adhesion, suggesting that some of these proteins are targeted to the cell membrane ( Huber & Sumper, 1994).

Lastly it is possible to take sequences belonging to one or several subtrees and align them using structural alignments. Using this approach, it is possible to get an indication of residues involved in substrate specificities or oxidation preference. 

## Conclusions

This is the first time that a phylogenetic tree showing both the intra- and inter-family relations of LPMOs is constructed. We believe that the new PRCx program will help researchers to determine where their LPMO is located in the phylogenetic tree, what the putative substrate specificities are and identify LPMOs with a yet unknown substrate specificity (e.g. the LPMO16s). Moreover, the PRCx program can also be applied to other large proteins families in which it can aid in discovering long distance evolutionary relations.

## Data availability

### Underlying data

All data underlying the results are available as part of the article and no additional source data are required.

### Extended data

Zenodo: Profile Comparer Extended: phylogeny of LPMO families using profile hidden Markov model alignments. http://doi.org/10.5281/zenodo.3518352 ( Voshol *et al*. 2019a).

This project contains the following extended data:

- Figure S1 (searchable phylogenetic tree).
- Table S1 (sequence data used in this study).

Data are available under the terms of the Creative Commons Attribution 4.0 International license (CC-BY 4.0).

## Software availability

**Source code for the PRCx program is available from:** https://github.com/gerbenvoshol/PRCx.

**Archived source code at time of publication:** http://doi.org/10.5281/zenodo.3518337 ( Voshol *et al*, 2019b).

**License:** GNU General Public License version 2.
