# Supplementary material for: Profile Comparer Extended: phylogeny of lytic polysaccharide monooxygenase families using profile hidden Markov model alignments
Source: F1000Res. 2019 Oct 31;8:1834. [Version 1] doi: 10.12688/f1000research.21104.1 (PMC6950343; doi:10.12688/f1000research.21104.1)
Supplement: Peer review history

# Peer review: Reviewer response for version 1

**DOI:** 10.5256/f1000research.23227.r57830

**Published:** 2020-01-08

**Authors:** Mirjam Kabel

**Affiliations:** 1 Laboratory of Food Chemistry, Wageningen University & Research, Wageningen, The Netherlands

The research describes the development of a sequence-model (profile hidden Markov model), which can be used to give insights into the inter- and intra-family relationships of protein families – based on their structure. The provided tool is, in particular, faster than the conventional tools to assess phylogenetic relationships.

 A phylogenic tree of over 10000 LPMO sequences was made to illustrate the usefulness of the LPMO-model developed. The authors show that their model indeed shows the expected AA families as listed in the CAZy database. But, what is less clear for me, is how could this LPMO-model be used to make phylogenic trees within one AA family? The authors mention that their model has only a length of 165, starting from the N-terminal histidine. Others (various papers, please check) have shown that LPMO-segments important for substrate recognition, all neighboring the active site, are spread over the total sequence. Hence, using the 165 amino acids only would lack information of certain binding-segments. Do I understand correctly that such information cannot be picked up? I would appreciate if the authors can better highlight for which applications their pHMM-approach could be used and for which it would not be suitable.

 As far as I can judge, still, the tool is an asset to make complex and more complete phylogenetic trees (more sequences) in a relatively short time. The research shown is relevant.

 Overall, the manuscript is clearly written, although several choices made could have been explained better (see itemized points below).

- Abstract: please specify "speeds several orders of magnitude" to be more specific.
- Abstract: remove the last sentence, it is not relevant. Or explain.
- Intro: "aid breakdown by conventional enzymes". Not absolutely clear, please reformulate.
- Intro: "sequence similarity between LPMO families". The sequence similarities within for example the AA9 family is also rather low even. Add?
- Results: phylogenetic tree: The authors refer to a paper in which phylogenetic trees per AA family are described, and subsequently write that this approach was not sensitive enough to show the relationship between the different families. Are these two not based on completely different research questions? Instead of being not sensitive enough, could the authors rewrite this section to better highlight the value of trees within and between AA families and which tools/models are best to use?
- Figure 5: for clarity, the y-axes could be converted to minutes.
- Figure 6: the model seems to pick up well the large AA families (AA9 and AA10), but in some of the others extra branches appear (i.e. AA16 & LPMO16). Is this a ‘real’ branching or the result of using the short 165 amino acid model? Please discuss and explain the consequences in the manuscript.

I confirm that I have read this submission and believe that I have an appropriate level of expertise to confirm that it is of an acceptable scientific standard, however I have significant reservations, as outlined above.

# Peer review: Reviewer response for version 1

**DOI:** 10.5256/f1000research.23227.r57829

**Published:** 2019-12-18

**Authors:** Stjepan Krešimir Kračun

**Affiliations:** 1 Chr. Hansen A/S, Hørsholm, Denmark

The authors have developed a UNIX-based program to study the phylogeny of large groups of proteins focusing on LPMOs. The program is novel, performs faster and contains features that no other program currently contains.

 The manuscript is written well, everything is clearly, accurately and sufficiently explained. 

 The methods used, prior art and references are addressed appropriately. 

 Specific comments and questions:

- LPMOs are a very interesting group of enzymes that have emerged as essential in degradation of biomass. 

The authors mention this briefly in their Introduction section (first paragraph).

 How do the authors envisage the utility and impact of their program for people who work with LPMOs in the wet-lab (enzyme discovery and screening, for example)?

 This should be reflected upon in the Conclusions section.

- The authors mention development of the program for UNIX-based platforms such as Linux and MacOS. Have they considered, or plan to implement an online solution? Without an online solution - the usefulness of the program will be severely limited. In addition, informing readers about how and when such a tool will be implemented, with a provisional online link (even if the implementation is not entirely completed) - will certainly increase the impact of the article.

I understand that most Linux code can be run on virtual machines, but from my experience - this is not necessarily accessible to people that are not bioinformaticians. Therefore, in order to make this tool useful for the scientific community - a user-friendly online solution would be highly recommended.

 Could the authors comment, as well as include in the manuscript, their plans regarding such implementation?

I confirm that I have read this submission and believe that I have an appropriate level of expertise to confirm that it is of an acceptable scientific standard, however I have significant reservations, as outlined above.

# Peer review: Reviewer response for version 1

**DOI:** 10.5256/f1000research.23227.r56318

**Published:** 2019-11-19

**Authors:** Miaomiao Zhou

**Affiliations:** 1 School of Life Sciences, Avans University of Applied Sciences, Breda, The Netherlands

The authors have developed a program to study the phylogeny of large groups of proteins. This new method, named PRC Extended (PRCx), was applied on the lytic polysaccharide monooxygenase (LPMO) from over seven families. Conventional pHMM-pHMM methods failed to perform such a large construction due to the overwhelming calculation requirements. In this study, the authors used both pHMM-sequence searches and pHMM-pHMM alignments, they extended the original Profile Comparer program (PRC) and added several capabilities. It is plausible that the authors updated the popular original PRC program with the ability to load HMMer3.0 pHMM files, this is a very desired feature among the users of the original PRC program.

 The authors applied PRCx on the large LPMO superfamily to study the inter and intra-family enzyme evolutionary relationship. This study was not feasible without the newly constructed PRCx program due to the large size of the superfamily. The PRCx program was able to build the phylogenetic tree for hundreds of HMMs within a few days, which is a huge improvement comparing to the original pHMM-based method.  

 During the preparation of the phylogenetic studies, the authors identified the PFAM LPMO_10 (PF03067) and GH61 (PF03443) families to be the in-groups. They also found PFAM Egh16-like family to be a distant relative of the LPMO family. They found the evidence to correct the mis-predicted cleavage site of the Egh16-like family PFAM HMM and used this family as the out-group for the study. The close examination of the resulting tree revealed several interesting features of the LPMO family. Many of these features point to potential experimental targets, for example, altering arginine, lysine and histidine in AA9 LPMOs might give impact on the activity level of the enzyme.

 Last but not the least, it is interesting to notice that the core LPMO pHMM has a model length of merely 165. Would a much bigger pHMM model compromise the performance of PRCx? Considering the fact that the authors tested the PRCx program on a moderate computer of Intel Core i5 with 8 GB of memory, a high performance computer with more RAM might solve the problem? 

 As a researcher in the fields of applied Bioinformatics in biotechnology, I would recommend PRCx, together with the phylogenetic analysis of LPMO, to be published. The PRCx program will be a very useful tool to study big enzyme groups beyond the LPMO superfamily.

 *availability: The authors have made their program available via github, the supporting data is all accessible via Zenodo.

I confirm that I have read this submission and believe that I have an appropriate level of expertise to confirm that it is of an acceptable scientific standard.
